# Preclinical Research of Dihydromyricetin for Brain Aging and Neurodegenerative Diseases

**DOI:** 10.3389/fphar.2019.01334

**Published:** 2019-11-11

**Authors:** Hilda Martínez-Coria, Martha X. Mendoza-Rojas, Isabel Arrieta-Cruz, Héctor E. López-Valdés

**Affiliations:** ^1^Division de Investigación, Facultad de Medicina, Universidad Nacional Autónoma de México (UNAM), Ciudad de México, México; ^2^Laboratorio Experimental de Enfermedades Neurodegenerativas, UNAM-INNyN, Instituto Nacional de Neurología y Neurocirugía “Manuel Velasco Suárez”,Ciudad de México, Mexico; ^3^Unidad Periférica de Neurociencias, UNAM-INNyN, Instituto Nacional de Neurología y Neurocirugía “Manuel Velasco Suárez”,Ciudad de México, Mexico; ^4^Departamento de Investigación Básica, Instituto Nacional de Geriatría,Ciudad de México, Mexico

**Keywords:** dihydromyricetin, ampelopsin, aging, neurodegenerative diseases, Alzheimer’s disease, Parkinson’s disease, Huntington’s disease

## Abstract

Brain aging and neurodegenerative diseases share the hallmarks of slow and progressive loss of neuronal cells. Flavonoids, a subgroup of polyphenols, are broadly present in food and beverage and numerous studies have suggested that it could be useful for preventing or treating neurodegenerative diseases in humans. Dihydromyricetin (DHM) is one of the main flavonoids of some Asian medicinal plants that are used to treat diverse illness. The effects of DHM have been studied in different *in vitro* systems of oxidative damage and neuroinflammation, as well as in animal models of several neurodegenerative diseases, such as Alzheimer’s disease, Parkinson’s disease, and Huntington’s disease. Here we analyzed the most important effects of DHM, including its antioxidant, anti-inflammatory, and neuroprotective effects, as well as its ability to restore GABA neurotransmission and improve motor and cognitive behavior. We propose new areas of research that might contribute to a better understanding of the mechanism of action of this flavonoid, which could help develop a new therapy for aging and age-related brain diseases.

## Introduction

Aging is a major risk factor for developing brain illnesses such as Alzheimer’s disease (AD) (Lindsay et al., 2002; Katz et al., 2012), Parkinson’s disease (PD) (Collier et al., 2011), and other age-related dementias (López-Valdés and Martínez-Coria, 2016), as well as cerebrovascular disorders (Choi et al., 1998). These diseases share pathophysiological mechanisms with aging such as oxidative stress and chronic inflammation.

Dihydromyricetin (DHM), also known as ampeloptin, rac-ampelopsin, and ampelopsin, is a flavonoid isolated mainly from Japanese raisin trees (*Hovenia dulcis* Thum) and Chinese Rattan tea [*Ampelopsis grossedentata* (Hand.-Mazz.) W.T.Wang]. These plants have been used for a long time in Asian traditional medicine to treat different health problems (Hyun et al., 2010; Kou and Chen, 2012). DHM is one of the main flavonoids found in the Japanese raisin tree and Chinese Rattan tea, together with myricetin and quercetin (Zhang et al., 2007; Hyun et al., 2010).

In the few past decades, scientific knowledge about the effects of DHM has increased considerably. This molecule has a wide range of positive effects, including anti-oxidative, anti-inflammatory and neuroprotective properties, and has been shown to cause motor and memory improvements, all of which can help treat dysfunctions associated with brain aging and some neurodegenerative diseases such as AD and PD.

This work summarizes and analyzes the current scientific information on the pharmacology of DHM, with a particular focus on the pathological processes of age-related brain diseases and on animal models of these diseases. We suggest some areas of research in which this compound could be shown to beneficial effects on human aging and age-related diseases.

### DHM Structure, Pharmacokinetics and Toxicity

Dihydromyricetin (2*R*,3*R*)-3,5,7-trihydroxy-2-(3,4,5- trihy droxyphenyl)-2,3-dihydrochromen-4-one is a flavanonol, a subgroup of flavonoids (PubChem CID: 161557 and ChEBI: 28429). This subgroup is a class of secondary plant metabolites that perform many physiological functions in plants as antioxidants, pigments, etc. (Tsao, 2010).

The pharmacokinetic parameters of DHM have so far been identified in rats after oral administration, the results indicating that DHM is poorly absorbed into the bloodstream, with a bioavailability of only 4.02%. Furthermore, the time required for it reaches peak plasma concentration is 2.67 h after oral administration at a dose of 20 mg/kg (Liu et al., 2017). A recent *in vitro* study using the human intestinal Caco-2 cell model, a common tool used to predict, *in vivo*, the absorption of drugs in humans, showed that the uptake and transport of DHM occurs mainly through a passive diffusion mechanism, which can partially explain the low bioavailability of DHM after oral administration (Xiang et al., 2018).

After ingestion by animals, some DHM is metabolized in the gastrointestinal tract and liver, and the rest is absorbed into the bloodstream and is widely distributed throughout the body, including the heart, lungs, kidney, etc., even crossing the blood–brain barrier and spreading through brain tissue (Fan et al., 2017). DHM is completely excreted in urine and feces after 12 h. Seven to eight DHM metabolites have been identified in urine, feces, and blood (Fan et al., 2017), all of them produced by common metabolic routes such as dihydroxylation, methylation, glucuronidation, reduction, and isomerization (Zhang et al., 2007; Fan et al., 2017). Whether these metabolites have any pharmacologic effect is still unknown.

Although there have been few DHM toxicity studies, some important information has already been obtained. For example, the lethal dose 50% for oral administration in mice is >5 g/kg (Zhou and Zhou, 1996). At concentrations ranging from 150 mg/kg (500 mmol/L) to 1.5 g/kg (5,000 mmol/L), DHM did not cause any acute toxicity or had significant side effects on mice (Zhang et al., 2014), which suggests that DHM has very low toxicity (OECD, 2001a; OECD, 2001b); however, more *in vitro* and *in vivo* tests are needed to establish reliable safety limits.

Since low bioavailability limits the pharmacologic value, several research groups have been made diverse preparations with better solubility or permeability, mainly tested on the *in vitro* studies. For example, microemulsion (Solanki et al., 2012), nanoparticles (Ameen et al., 2018), soluble cocrystals (Wang et al., 2016), nanoencapsulation (Dalcin et al., 2019), and solid dispersions and inclusion complex (Ruan et al., 2005). Additionally, Zhao et al. (2019) showed that a nanoscale DHM-phospholipid complex significantly increased oral bioavailability in rats. All these developments can improves the oral bioavailability and facilitate their possibly use against brain aging and neurodegenerative diseases.

### Anti-Oxidative Effects of DHM (*In Vitro* Models)

Oxidative damage to cells is a common and important part of the pathology of many diseases such as AD, PD, Huntington’s disease (HD), and aging (López-Otín et al., 2013; Singh et al., 2019). The molecules responsible for oxidative damage, known as reactive oxygen species (ROS) and reactive nitrogen species (RNS), are mainly produced by the mitochondrial respiratory chain. Several *in vitro* studies have shown that DHM inhibits lipid-peroxidation (He et al., 2003a; He et al., 2003b; Zhang et al., 2003; Yang et al., 2009), which suggest that DHM can protect cell membrane lipids against the damage induced by an excess of ROS and RNS. Moreover, DHM may reduce oxidative damage to cells through different mechanisms such as direct radical-scavenging and Fe^2^-chelation (Li et al., 2016), as well as by increasing the enzymatic activity of superoxide dismutase (SOD), which mainly catalyzes the dismutation of superoxide anion (O2•–) to molecular oxygen (Mu et al., 2016; Song et al., 2017). Moreover, DHM also exerts antioxidant effects by activating phosphatidylinositol 3-kinase (PI3K/Akt) and modulating the nuclear transcription factor-erythroid 2-related factor 2 (Nrf2), which participates in the induction of genes encoding detoxifying and antioxidant enzymes (Luo et al., 2017; Hu et al., 2018). Another study suggests that DHM modulate AMP-activated protein kinase to cause inhibition of the oxidative stress response (Jiang et al., 2014). Taking all the above into account, DHM seems to reduce the oxidative stress by modulating several molecules of this signaling pathway.

### Anti-Inflammatory Effects of DHM (*In Vitro* Models)

Although some aspects of the anti-inflammatory effect of DHM are still unknown, some *in vitro* studies have shown that it decreases the production of different molecules that are part of the pro-inflammatory cascade, such as interleukin IL-1β and IL-6, the tumor necrosis factor-alpha (TNF-α) and nitric oxide (Qi et al., 2012; Hou et al., 2015), while also increasing the production of anti-inflammatory IL-10. Moreover, DHM downregulates the expression of TNF-α by inhibiting nuclear factor-kappa B (NF-kB), a protein complex that controls cytokine production and is involved in many processes, including inflammation and apoptosis (Tang et al.,2016). Moreover, another research found that DHM decrease dysfunction (tube formation and migration) of Human Umbilical Vein Endothelial Cells (HUVECs) Culture, induced by TNF- α through repressing miR-21 expression (Yang et al., 2018), a microRNA which increased expression was observed in human atherosclerosis (Raitoharju et al., 2011).

### Effects of DHM in Animal Models of Aging

A common model for the study of aging is the D-galactose-induced aging rats, produced by the chronic administration of D-galactose (D-gal), which causes mitochondrial dysfunction and increases apoptosis, inflammation and oxidative stress in the brain. These animals show alterations such as poor immune response, a shortened lifespan, and learning and memory deficits (Shwe et al., 2018). In this animal model, DHM has been shown to cause a significant reduction astrogliosis, apoptosis and dysfunctional autophagy in neurons of the hippocampus through the up-regulation of sirtuin 1 (SIRT1) and p53/p21, and the down-regulation of the mammalian target of rapamycin (mTOR) in a miR-34a-dependent manner (Kou et al., 2016). These results suggest that DHM ameliorate cognitive impairments and aging by modulate excessive apoptosis and dysfunctional autophagy of hippocampal neurons, and astrogliosis.

### Effects of DHM in Animal Models of AD

A few years ago we published a study (Liang et al., 2014) about the chronic effects of DHM in two different transgenic mouse models of AD (TG2576 and TG-SwDI). We used 20 months-old male animals divided into transgenic and control groups. The animals were treated with 2mg/Kg/day during 3 months and we analyzed the effects of DHM on their behavior and pathology. The results showed that chronic treatment with DHM improves memory, decreases the accumulation of Aβ1–40 and Aβ1–42 (markers for AD), and restores Gamma-Aminobutyric Acid (GABA) neurotransmission and the levels of gephyrin, an anchor protein for the postsynaptic GABAA receptor. These results suggest that chronic treatment with DHM improves cognitive deficits, reverses the progressive accumulation of Aβ peptides, and restores GABAergic transmission and functional synapses. Recently, Feng et al. (2018) used another Alzheimer’s mouse model, termed APP/PS1 double-transgenic, in which the mouse/human amyloid precursor protein and the mutant human presenilin-1 are overexpressed. They showed that DHM treatment also improves memory and decreases the number of activated microglia and the NLRP3 inflammasome, the activation of which plays an important role in chronic brain neuroinflammation (Heneka et al., 2018). Another recent study (Sun et al., 2019) done in an AD rat model induced by intracerebroventricular injection of Aβ1–42, found that DHM (100 and 200 mg/kg for 21 days) improves learning and memory, decreased hippocampal neuronal apoptosis, IL-1β, IL-6, and TNF-α in serum and hippocampus and changes in different proteins such as decreased pro-apoptotic Bax and NF-κB and increased anti-apoptotic Bcl-2, pAMPK, AMPK, and SIRT1, suggesting that beneficial effects of DHM are mediated by up-regulation of AMPK/SIRT1 pathway.

### Effects of DHM in Animal Models of PD

A common animal model of PD is based on the administration of the protoxicant MPTP (1-methyl-4-phenyl-1, 2, 3, 6-tetrahydropyridine) for several days. After MPTP crosses the blood-brain barrier, it is metabolized by glial cells, which produce and release the toxic metabolite MPP+(1-methyl-4-phenylpyridinium ion), which is selectively transported into dopaminergic neurons of the substantia nigra pars compacta, where it reduces ATP production and increases the production of ROS (Blandini and Armentero, 2012). Recently, Ren et al. (2017) used the MPTP animal model of PD to study the effects of DHM and found that the administration of DHM for thirteen days (tree days before the start of MPTP, seven days during MPTP administration and three days after the end of MTPT) significantly decreased dopaminergic neuronal loss and motor impairments (evaluated using the climbing pole and rotarod tests) by a mechanism that involves the inhibition of ROS production as well as a reduction in the production of the toxicant MPP+ through the inhibition of glycogen synthase kinase-3β (GSK-3β), the activity of which is associated with dopaminergic neuronal death caused by MPTP and by the neuroinflammation process associated with PD (Golpich et al., 2015).

### Effects of DHM in Animal Models of HD

In a 3-NP rat model of HD, treatment with DHM (10mg/kg/day for five consecutive days) reduced significantly the initial and total time in the balance beam task, the hang time in the grip-strength test, and escape latency and time in the target quadrant in the Morris water-maze test, all together suggest that DHM reduced motor, learning, and memory impairments. Moreover, DHM increased the striatal metabolic rate, and reduced oxidative stress and apoptosis. The mechanisms behind these effects include the down-regulation of the pro-apoptotic Bax protein and the up-regulation of the anti-apoptotic Bcl-2 protein, increased SOD activity and decreased Malondialdehyde, a final product of polyunsaturated fatty acids peroxidation (Mu et al., 2016).

### Last Considerations

It is worthwhile to note, that DHM it is not the only flavanonol that has been studied as a potential neuroprotective agent. Taxifolin, also known as Dihydroquercetin or Distylin (PubChem CID: 439533), shows neuroprotective effects such as reduction of Aβ1–40 and Aβ1–42 formation, improves cognition in a transgenic mouse model of cerebral amyloid angiopathy and in AD mouse model induced by injection of Aβ1–42 on the hippocampus (Davis et al., 2004; Sato et al., 2013; Saito et al., 2017; Wang et al., 2018). Although, DHM and Taxifolin have a similar molecular structure and they share some activation pathways (e.g. BDNF and SIRT1) more research is needed to establish a structure-activity relationship of flavanonols.

### Conclusions and Future Prospects

**Figure 1:**
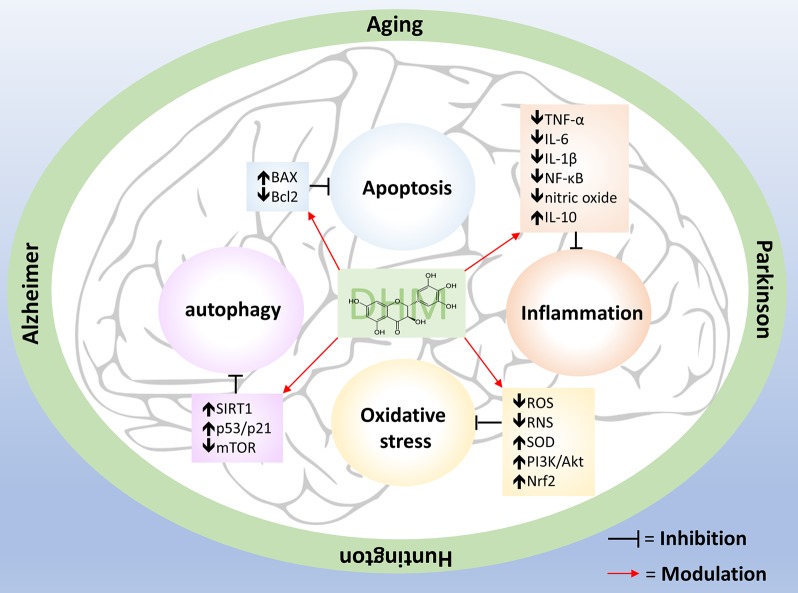
Pleiotropic effects of DHM. Aging, AD, HD and PD have several dysfunctional responses such as oxidative stress, chronic inflammation, apoptosis, and autophagy impairment. DHM counteract these alterations by modulate specific molecules (showed inside the boxes) that are part of those pathways.

In this review, we showed that DHM has a wide range of beneficial effects, both *in vitro* and *in vivo* models of human diseases, including anti-oxidative, anti-inflammatory and neuroprotective properties (**Figure 1**), restoration of GABA neurotransmission, and improvements in motor and cognitive behavior, which suggests that this compound may be useful for treating human aging and neurodegenerative diseases. However, further research is required to assess the clinical potential of DHM. For example: several studies have indicated that DHM is absorbed in the gastrointestinal tract, crosses the blood-brain barrier and does not have toxic effects, suggesting that it could exert positive effects on the brain without risk of toxicity; however, more pharmacokinetic studies are required to provide more robust evidence. Although we have a good understanding of some of the effects of DHM on cells found outside of the brain, we still do not understand completely the basic mechanisms of neuroprotection and neuroplasticity of DHM in neurons and glial cells, particularly in microglia and astrocytes, which play a very important role in the homeostatic maintenance of the brain. Besides this, senescent cells are also always present in aging and neurodegenerative diseases, and we need to know whether DHM can modulate cellular senescence and the inducers of this process. Anti-inflammatory effects of DHM have been broadly studied, mainly *in vitro* and *in vivo* models, and a clinical trial has shown some similar results. A randomized, double-blind, placebo-controlled trial in humans showed a significant decrease in seric TNF-α after 12 weeks of consuming 600 mg of DHM per day (Chen et al., 2015), supporting the idea that DHM has positive effects on human health.

## Author Contributions

Conceptualization, HM-C and HEL-V. Writing original draft preparation, HM-C, HEL-V, MM-R, and IA-C. Writing review and editing, HM-C, HEL-V, MM-R, and IA-C. Supervision, HEL-V and HM-C.

## Funding

This project was supported by a grant from the Secretaria de Educación, Ciencia, Tecnología e Innovación de la Ciudad de México SECITI/042/2018 (INGER-DI-CRECITES-002-2018 to IA-C) “Red Colaborativa de Investigación Traslacional para el Envejecimiento Saludable de la Ciudad de México (RECITES)”.

## Conflict of Interest

The authors declare that the research was conducted in the absence of any commercial or financial relationships that could be construed as a potential conflict of interest.
